# Better Medicines for Older Patients: Considerations between Patient Characteristics and Solid Oral Dosage Form Designs to Improve Swallowing Experience

**DOI:** 10.3390/pharmaceutics13010032

**Published:** 2020-12-28

**Authors:** Nélio Drumond, Sven Stegemann

**Affiliations:** Graz University of Technology, Inffeldgasse 13/III, 8010 Graz, Austria

**Keywords:** swallowing problems, dysphagia, older patients, solid oral dosage forms, administration aids, administration devices, film coating materials, patient centric drug product design

## Abstract

Oral drug administration provided as solid oral dosage forms (SODF) remains the major route of drug therapy in primary and secondary care. There is clear evidence for a growing number of clinically relevant swallowing issues (e.g., dysphagia) in the older patient population, especially when considering the multimorbid, frail, and polymedicated patients. Swallowing impairments have a negative impact on SODF administration, which leads to poor adherence and inappropriate alterations (e.g., crushing, splitting). Different strategies have been proposed over the years in order to enhance the swallowing experience with SODF, by using conventional administration techniques or applying swallowing aids and devices. Nevertheless, new formulation designs must be considered by implementing a patient centric approach in order to efficiently improve SODF administration by older patient populations. Together with appropriate SODF size reductions, innovative film coating materials that can be applied to SODF and provide swallowing safety and efficacy with little effort being required by the patients are still needed. With that in mind, a literature review was conducted in order to identify the availability of patient centric coating materials claiming to shorten esophageal transit times and improve the overall SODF swallowing experience for older patients. The majority of coating technologies were identified in patent applications, and they mainly included well-known water soluble polymers that are commonly applied into pharmaceutical coatings. Nevertheless, scientific evidence demonstrating the benefits of given SODF coating materials in the concerned patient populations are still very limited. Consequently, the availability for safe, effective, and clinically proven solutions to address the increasing prevalence of swallowing issues in the older patient population is still limited.

## 1. Introduction

The improvements in modern healthcare provision, combined with the availability of new effective drug therapies, are both contributing to a continuous increase in average life expectancy [1]. Ageing is associated with an increasing incidence of chronic diseases and co-morbidities, which leads to the practice of polypharmacy amongst the majority of the older patients [2]. This topic raises safety concerns, as it was previously reported that a least 16.5% of older patients under polypharmacy regimens have gone through hospitalization, or even death, as an outcome of medication-related issues (e.g., drug interactions) [3,4].

The oral route is considered to be, by far, the most preferred and convenient for the majority of patients, as it is non-invasive in, and allows for, independent usage and handling [5,6]. Nevertheless, one must consider that the swallowing function in older patients is expected to be impaired, due to ageing and chronic conditions (dysphagia), which may raise challenges to swallow solid oral dosage forms (SODF) effectively and safely [7,8]. Swallowability and palatability are attributes that impact the acceptability of SODF by patients [9,10,11], which can be affected by the SODF physical properties upon deglutition and esophageal transit time [1,9,12,13,14,15,16,17,18,19,20,21,22,23,24]. As such, more effort needs to be put into the design of patient centric drug products that can benefit older patients and their experience with prescribed medicines [8,25,26].

This literature review provides an overview on physical characteristics of older patients that can impact the administration and acceptability of drug therapies that are provided in SODF, including their relation to specific SODF designs. Descriptions of conventional techniques, swallowing aids, and administration devices targeting this special patient population in order to improve their swallowing experience with SODF are also given. Moreover, the importance of using a patient centric drug product design approach when developing appropriate SODF for the older patient population is also discussed, being supported by a literature review on the film coating materials designed to enhance the swallowing experience and acceptability of SODF for older patients with impaired swallowing functions, including their clinical evidence for improved efficacy and safety.

## 2. Swallowing Problems in the Older Patient Population

Dysphagia is a growing concern for the health of older and multimorbid patient populations, as it tends to remain an underestimated symptom [27,28,29]. Previous findings suggested that 46% of patients with dysphagia do not inform their doctor regarding their condition, while 70.4% of patients are not properly diagnosed as having dysphagia [30]. In addition, patients report that their pharmacists and doctors rarely inquire about their swallowing function [31,32]. Therefore, it is important that healthcare professionals question older patients regarding their swallowing function (and rule out dysphagia as a symptom) in order to ensure that appropriate solid dosage form designs are being provided [32,33,34,35].

### 2.1. Prevalence of Dysphagia

Swallowing problems are predicted to affect one out of 25 adults. Previous surveys have identified that approximately 9.5 million adults (mean age: 52.1 years) report swallowing problems yearly, with women being more likely to report the problem as compared to men. In USA, it is expected that more than six-million older adults experience swallowing issues [11,36]. Other reports have suggested that more than 15% of the older population suffers from dysphagia worldwide, from which only 22.7% visited their healthcare professional in order to address the condition [35]. Therefore, a continuous growth is expected in the prevalence of swallowing disorders regarding older patients, as life expectancy is expected to increase in the future.

### 2.2. Factors Contributing to Dysphagia

There are many reasons and underlying etiologies that contribute to the development of swallowing problems. These can be classified into age-related, disease-related, and drug-related dysphagia.

#### 2.2.1. Age-Related Dysphagia

Age-related changes in the swallowing physiology are predisposing factors for dysphagia in the older patient population [19,37]. These are typically related to anatomic, motoric, and sensory alterations, which become less efficient when responding to the body stimulus and they lead to a subtle decay in the swallowing function with increasing age [33,38,39]. The diagnosis of dysphagia in older patients usually remains asymptomatic and it only becomes visible in advanced stages of deterioration or when associated to other clinical conditions [1].

#### 2.2.2. Disease-Related Dysphagia

Dysphagia can also develop as a co-morbidity, due to an increasing incidence of chronic conditions or disease-specific patterns in older patients (Table 1). Examples include neurological disorders and neurological damage (e.g., Parkinson’s disease, Alzheimer’s disease, dementia, multiple sclerosis, muscular dystrophy, stroke, and spinal cord injury), chronic obstructive pulmonary disease, congestive heart failure, and xerostomia [13,14,15,16,17,18,19,20,21,30]. Furthermore, conditions that impact the swallowing reflex (e.g., osteoarthritis, thyroid disease, hypertension, hypercholesterolemia, gastroesophageal reflux, and depression) may also predispose patients to dysphagia, due to their association with prolonged pharyngeal and oropharyngeal transit times upon swallowing [22,23].

#### 2.2.3. Drug-Related Dysphagia

Patients with long-term exposure to certain classes of drugs are more susceptible to developing swallowing problems as a result of their pharmacological activity, the likelihood of adverse drug reactions (ADRs), and medication-induced esophageal injury [24]. ADRs are usually associated with drugs that affect the smooth/striated muscle function [40]. Immunosuppressive drugs, antineoplastic agents, and antibiotics have been identified to increase the incidence of dysphagia as a complication of its pharmacological effects [41]. Finally, esophageal injury can also be induced by medications that have a direct erosive effect in the mucosa (dose dependent) or an indirect modification of the physiological pH of the esophagus [42]. Some examples include anti-infective drugs (e.g., tetracyclines, penicillin, and macrolides), steroidal anti-inflammatory drugs (e.g., piroxicam, acetylsalicylic acid), emepronium bromide, and quinidine (Table 2). Medication-induced dysphagia is expected to be one of the leading etiologies for esophageal motility disorders in older patients [23,43,44].

### 2.3. Perception of Dysphagia by Older Patients

The extent to which older patients are aware of a possible deterioration of their swallowing function remains unknown. Some findings point out that patients experience an impairment in swallowability; however, it is unclear how they perceive this [38]. Discrepancies between patient complaints and objective swallowing diagnosis have been reported, while positive associations were identified in other studies [45,46,47,48,49,50]. Notwithstanding, one significant correlation has been pointed out, which is related to a reported difficulty in swallowing by the patients and their measured swallowing efficiency values [51].

## 3. Administration of SODF by Older Patients

**Figure 1:**
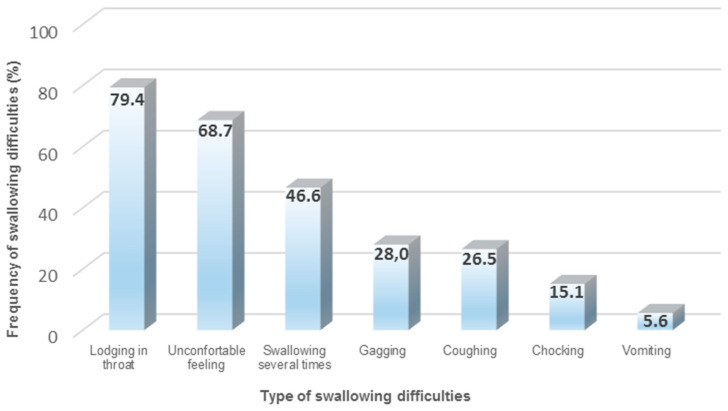
Common types of swallowing difficulties when administering solid oral dosage forms (SODF) [34].

The majority of available drug therapies on the market are SODF (65–70%), such as tablets and capsules with different sizes and shapes. SODF remain very popular for manufacturing companies, due to different reasons (e.g., cheap manufacturing, accurate dosing, patient acceptability, and taste masking) [52,53,54]. However, when considering older patients and their incidence for polypharmacy, the administration of SODF can become a daunting task [55,56]. Previous research has identified that one in three patients experience situations of vomiting, gagging, or choking when administering SODF (Figure 1). Furthermore, it has been noted that, during SODF administration, older patients with dysphagia demonstrate longer swallowing times, a higher number of swallows, and the need of water to support the SODF bolus [57]. The combination between impaired swallowing function and poor dosage form design (e.g., large round tablets) may contribute to an unpleasant patient experience, due to potential adherence or lodging of the SODF in the esophagus, reducing the acceptability and compliance for prescribed treatments [34,58,59,60,61]. Subsequently, older patients cope with the situation by either skipping doses or modifying the SODF (e.g., crushing and splitting tablets, opening capsules) for an easier swallowing experience [62,63,64,65,66]. 

SODF modifications are seen as the most common technique that is used by older patients and their caregivers to improve the. administration of SODF [12]. A survey in Germany showed that 58.8% of dysphagia-affected patients manipulate their drugs for easier administration [34]. Dosage form modifications should be avoided if not specified in the drug product label. Improper manipulations can endure the unpleasant taste of masked ingredients and modify the controlled release properties, which can lead to poor efficacy or clinically relevant ADRs [67,68,69].

### 3.1. Conventional Administration Techniques to Improve Swallowability

**Figure 2:**
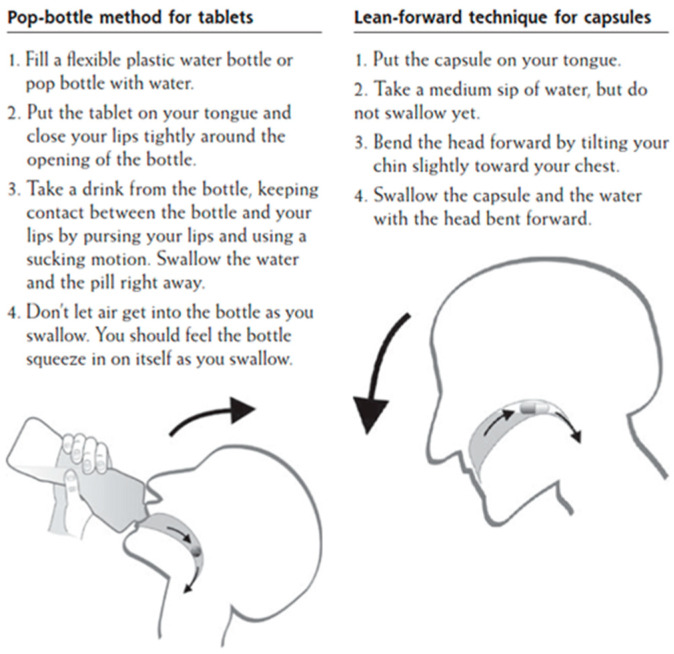
Patient information handout on conventional techniques to swallow SODF [72].

A study that was conducted in Germany investigated the efficacy of swallowing large tablets and capsules by applying two distinct administration strategies. The “pop-bottle” method was applied in order to swallow large tablets, whereas the “lean-forward” technique was applied for large capsules (Figure 2). The “pop-bottle” is a method where the tablet is placed on the tongue, the lips are tightly closed around the opening of a plastic bottle, and the tablet is swallowed in a swift suction movement in order to overcome the initial, volitional step of the swallowing act [70]. In the “lean forward” technique, capsules are swallowed in upright position with the subject’s head bent forward [71]. The SODF were swallowed with 20 ml of water and the overall swallowing experience was evaluated through a questionnaire. The obtained results revealed that both of the techniques significantly improved SODF administration and, as such, this study was the first to demonstrate that conventional techniques for SODF administration can be adopted [72]. Nevertheless, these methods require training, and they are highly dependent on the patient’s characteristics, which may restrict their use in general practice. In addition, the approval to apply such administration techniques should first be confirmed first by a physician, as there is an expected risk of aspiration considering older patients with dysphagia [73].

Other studies have shown that body position can influence the esophageal transit time of tablets, which confirms that a correct body posture must be adopted when administering SODF [74]. Longer transit times were observed for patients taking SODF in supine position as compared to the upright position. This is a matter of concern for bedridden patients, as these may be subjected to esophageal injury, due to slower transit times regarding the SODF taken [75,76]. 

### 3.2. Application of Administration Aids and Devices to Improve Swallowability

#### 3.2.1. Oral Jellies

**Figure 3:**
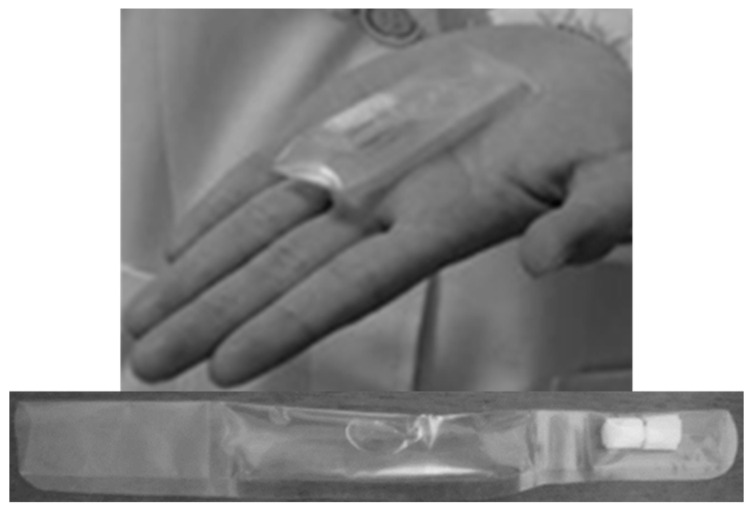
Packaged jelly formulation to aid tablet swallowing [83].

Food aids with semi-solid consistency such as oral jellies, are commonly applied as an administration vehicle by older patients, because their rheological properties allow for the formation of a bolus that incorporates the SODF and promotes a better swallowing experience [77,78,79,80,81]. Different reports have shown that the use of viscous oral jellies in the replacement of water tend to reduce the cases of aspiration and choking with large SODF for older patients with dysphagia [82]. Another study in Japan investigated the applicability of a swallowing aid that consists of two sections: an upper part containing the SODF to be swallowed and a bottom part, including an amount of oral jelly (e.g., xanthan gum) to support administration (Figure 3). The majority of the participants agreed that the administration vehicle (GT packaging) was convenient and supported swallowability (Table 3) [83]. Intellectual property (Table 4) while using jelly-based administration vehicles to assist SODF administration have been also reported [84,85,86].

#### 3.2.2. Pill Glide^®^

**Figure 4:**
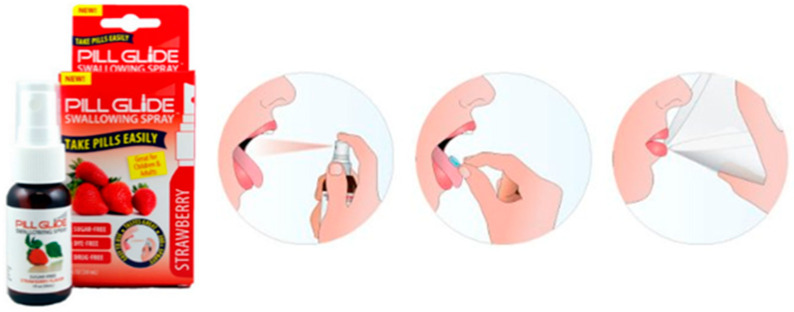
Schematic representation on how to use Pill Glide^®^ to aid swallowing of SODF [94].

A flavored spray was developed in order to provide a better experience during swallowing of SODF (Figure 4). The spraying of *Pill*-*Glide^®^* into the mouth and tongue of the patient generates a mucosa-coated surface that becomes slippery and later facilitates the swallowing of the SODF [92,93]. In a clinical assessment (Table 3), *Pill Glide^®^* improved the SODF swallowing experience in adolescents [87]. Although data is only reported for young patients, the product is recommended to people of all ages that struggle with SODF swallowability, including older patients [94]. A patent disclosing an anti-stick formula that is delivered by spray (Table 4) in order to facilitate swallowing is also reported [89].

#### 3.2.3. SODF Coating Devices

##### MedCoat^®^

**Figure 5:**
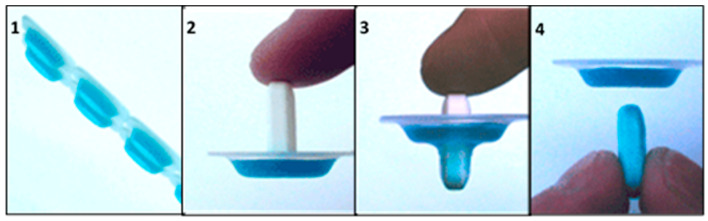
Schematic representation on how to apply MedCoat^®^ onto SODF [95].

MedCoat^®^ is an administration aid device that was designed to allow patients to independently apply coatings to their SODF before swallowing (Figure 5). The coating contains maltitol (sweetener), vegetable fats (coconut and palm oils), gelatin, sugar esters of fatty acids (emulsifiers), citric acid, and lemon flavor additives for taste masking and saliva stimulation. The coating is applied by passing the tablet through a ring that is covered by a gelatinous film before administration [95]. A clinical trial that was conducted in Lithuania (Table 3) has shown that SODF coated with MedCoat^®^ were easier to swallow for older patients presenting swallowing issues [88]. A patent disclosing this technology (Table 4) was reported in 2010 [90].

##### Coating Container

**Figure 6:**
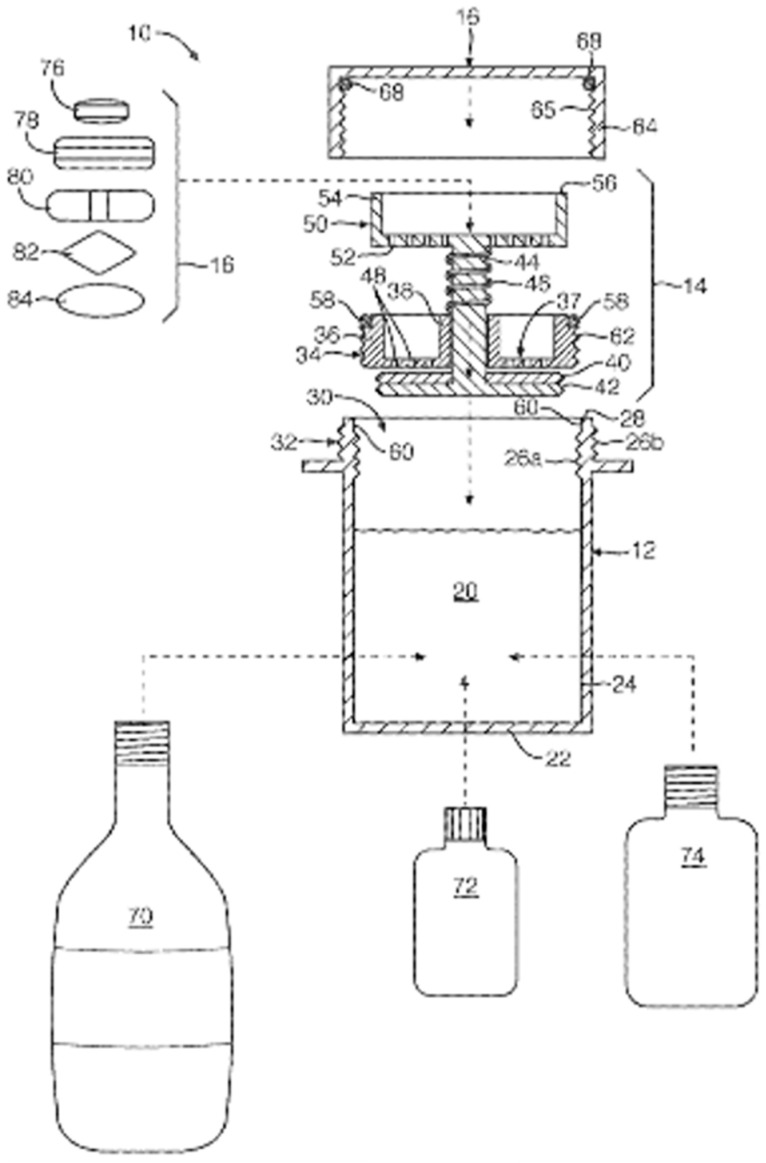
Schematic representation of container to coat SODF [91].

A vessel system in which the SODF can be inserted and coated was developed (Figure 6). The vessel system is composed of the container, contained cap, and internal closure assembly. The container can be filled with a coating liquid that is sealed by the closure assembly and cap [91]. The SODF are fitted between the cap and valve closure assembly, followed by the fitting of the closure assembly on the container. The coating liquid is composed of vegetable oils, surfactants, and flavoring agents that alter the surface properties of the SODF, thus improving swallowability (Table 4). 

### 3.3. Influence of SODF Design on Patients’ Adherence and Swallowing Experience

Previous reports detailed that the adherence to self-administering drug therapies is around 50%, with the decrease being related to an increased complexity, inconvenience, or duration of the regimen [96]. Another study identified swallowability as being the most important characteristic of SODF for improving acceptability for older patients [97]. Swallowability and esophageal transit time can both be impacted by the physical attributes and technology-related characteristics of SODF. Physical attributes, such as tablet size, shape, thickness, color, and surface roughness, were strongly associated to medication adherence [67,98], from which tablet size, shape, and thickness were identified as critical attributes for proper handling and swallowability [99]. Technology-related characteristics of SODF, such as disintegration time, surface roughness (e.g., film coating), and propensity for swelling, were other important parameters that were also identified with impact swallowing performance [100,101].

#### 3.3.1. Color 

Specific SODF colors can be associated to taste and flavor by older patients. The pink color tends to be linked to sweet flavors, whereas yellow tablets can be perceived to have a salty taste, irrespective of formulation ingredients [102]. The color of SODF are an important criteria for patients with specific conditions (e.g., epileptic), since its modification can lead to cases of non-adherence [103]. Overall, the white color is recognized as the most popular choice for tablets, while the most disliked colors are purple and brown [97]. Although color appears to be of least importance for patient adherence, it is, on the other hand, considered to be the most distinctive and memorable attribute for a SODF [99].

#### 3.3.2. Size

A usual approach for increasing patient compliance and reducing pill burden is done by increasing the SODF size in order to accommodate a higher dose strength [104]. This rule does not apply to older patients, as these perceive SODF as being more difficult to swallow with increasing size and consider the size of the SODF to be the most important physical attribute for swallowing safety [26,100,102]. This is supported by a study that identified a correlation between higher esophageal muscle effort with an increasing size of the SODF to be administered. Other studies have also shown that larger SODF administered by elderly patients tend to present longer esophageal transit times [75,76,97,100,101,105,106]. With regards to handling and easiness of swallowing, a study that was conducted in Japan showed that 7–8 mm tablets were perceived to be the most desirable size for old frail patients [107].

#### 3.3.3. Shape

Several studies have evaluated the impact of different SODF shapes on older patients’ swallowing experience. Flat-shaped tablets were seen as being more likely to adhere to the esophagus when compared to convex-shaped tablets [100,106], whereas oval and oblong tablets have shown faster esophageal transits as compared to round tablets with the same density. The oblong shape was seen to be the preferred for SODF, as it was reported to provide a better administration experience regarding patients with swallowing issues [1,101,104]. The shape of SODF is also considered to be the most memorable characteristic for older patients, alongside the color [99]. 

#### 3.3.4. Taste and Smell

Previous studies have identified that the bad taste of SODF was the fourth major complaint of patients, behind size, surface, and shape [58]. Furthermore, cases of non-adherence have also been reported, due to the potential bad taste and smell of SODF [106]. Taste masking is a very common technique that is applied during granulation and coating processes, as there are many drugs with bitter taste (e.g., Ibuprofen) [108]. 

#### 3.3.5. Density

SODF with higher density typically were shown to present faster transit times when compared to similarly-sized tablets with less density [100]. Large and dense capsules are related with quicker esophageal transit times when administered by patients in an upright position, whereas capsules with lower densities exhibited the same profile when swallowed in the supine position [75]. A positive correlation between the density of capsules and their tendency to stick in the patient’s esophagus was also identified [106].

#### 3.3.6. Surface Characteristics

Several studies have assessed the impact of SODF coated surfaces and their relation to the patient swallowing experience. It was observed that coated tablets reduce the number of swallows and the strength of swallowing regarding patients with dysphagia. A higher esophageal contraction force was required by the patients in order swallow large uncoated tablets, whereas the presence surface coating in the SODF reduced their swallowing effort. The transit time was also reduced when a coating surface was present in the SODF [101]. A higher risk for the lodging of SODF in the esophagus was also identified for uncoated tablets when compared to the identical coated tablets [100]. The surface roughness of SODF may also increase their likelihood for sticking in the esophagus, leading to an unpleasant swallowing experience for older patients. The stickiness was also positively correlated to the SODF surface area, while the presence of SODF film coatings led to an improvement in their transit times [75]. Overall, the SODF coatings have demonstrated to considerably reduce the cases of non-adherence and SODF manipulation to enhance swallowability, and they should always be integrated into SODF product design [1,97,99,101].

## 4. Development of SODF for Older Patients Requires a Patient Centric Drug Product Design Approach

It is a general understanding regarding drug product design a “one size fits all” approach cannot address the specific needs of heterogeneous older patient populations worldwide [25,109,110,111]. Previous reports suggested that a tablet weight within 300–450 mg provides a good balance between the handling and swallowing experience [99]. However, such an approach only covers a limited number of drugs and it does not apply to SODF requiring a higher dose strength (e.g., 1000 mg tablets). It is generally perceived by older patients that a better swallowing experience can be achieved with coated SODF that are small, oblong, and strongly convex. In addition, for SODF requiring higher doses, the preferred shape tends to be oblong and/or oval [67,106].

New guidelines that were published by the European Medicines Agency (EMA) were implemented in order to encourage the development of drug product designs that can address the specific needs of older patients [112]. Nevertheless, although regulatory incentives have been initiated, the availability of SODF designs that can really benefit older patients are still lacking [113,114,115,116]. 

Recent developments in patient-friendly dosage forms were achieved with the development of orally disintegrating tablets (ODTs) [117,118]. These are easy-to-swallow dosage forms that disintegrate within seconds upon uptake of saliva in the mouth, and they can be therefore swallowed in the form of a liquid or suspension [119,120,121,122,123,124]. Notwithstanding, the administration of non-solid formulation can be associated with a higher risk for aspiration regarding dysphagic patients, as compared to conventional SODF [125,126,127].

As it is well understood that older patients struggle to swallow large SODF, a simple patient-centric approach could focus on the manufacturing of reduced dosage form sizes in order to enhance swallowing experience and patient compliance [26,128,129,130,131,132,133]. Following this concept, and for a given pharmaceutical drug product, a wide range of SODF presentations should be available on the market to meet the heterogeneous needs of the older patient population [134,135,136,137]. Examples may include not only minitablets [138,139,140,141] and multiparticulate systems [142,143,144,145,146], which are patient centric for supporting a better swallowing experience and flexible dosing [147,148], but also chewable tablets [149] and buccal films [150,151,152]. For cases of drug products that remain in a conventional SODF presentation (e.g., tablets or capsules), a patient-centric approach for addressing older patients could involve the development of appropriate film coating materials that can contribute for faster transit times and reduce their likelihood to stick or lodge in the esophagus [153,154,155,156]. New non-mucoadhesive film coating materials that exhibit enhanced gliding performance throughout the oro-esophageal system are still required to address this [157,158,159].

## 5. Film Coating Materials Designed to Enhance SODF Swallowing Experience

A literature review on available scientific articles and patents that described film coating materials (and their polymer compositions) targeting swallowability enhancement for SODF was performed in May 2015 by an experienced librarian while using established methodology [160]. A list of suitable keywords (Supplementary material, A) and relevant truncations were developed in order to support the search (Supplementary data, B) using different search engines (e.g., Scifinder, Web of Science, Medline). The patents were searched by using the self-programmed Retrieval-Engine available from Espacenet. In December 2020, a complementary literature review was conducted while using PubMed database to update the search strategy regarding the time frame between 2015 and 2020. All of the searches were performed with no date of publication, language, or geographic restrictions. The term “palatability” was not included in the search strategy in order to avoid biased results representing patient acceptability with regards to flavoring agents (e.g., taste) because the main focus of the review was targeted on swallowability enhancement.

### 5.1. Selection Process and Obtained Results

The authors (DRU, STE) independently performed a primary screening by reviewing the title and abstract for the retrieved publications. Articles with no relevant content, as decided by the two authors, were eliminated from the search result. The full text of the remaining articles was individually reviewed and screened according to pre-established inclusion and exclusion criteria (Table 5). The resulting publications were analyzed and evaluated by the authors for their research target, research methodology, and data interpretation.

The combined literature searches that were performed at the different time frames using the relevant databases and search criteria resulted in 425 citations. The preliminary examination of their potential relevance led to the exclusion of 282 references. Publications that were related to the remaining 143 citations were screened while using the established inclusion and exclusion criteria. This resulted in the exclusion of 113 citations, with the remaining 30 references being included in the review. From the included references, two were scientific articles (Table 6) and twenty-eight were patents (Table 7). It is worth noting the limited availability of scientific articles, which contrasts with the large number of patent applications aiming at retaining intellectual property and reducing competitiveness, as the formulations that are employed to manufacture SODF coatings are composed of well-known polymers that are present in the market for decades.

#### 5.1.1. Polyvinyl Alcohol-Based Coatings (PVA)

Researchers in Japan developed a swellable tablet coating that was composed of PVA and carboxyvinyl polymer [161]. A patent application has also been disclosed for this technology [184]. Another two patents have also described PVA combinations with polyacrylic acid/ glycerin and guar gum/triglycerides, respectively [178,190].

#### 5.1.2. Cellulose-Based Coatings

Different hydroxypropyl methylcellulose films (HPMC) were suggested alone [176], and in combinations with triacetin [165] or ethyl cellulose (EC)/polyvinylpyrrolidone (PVP) [167].

#### 5.1.3. Gum-Based Coatings

Coating materials comprising gum arabic in association with gelatin [163] and sodium alginate/methylcellulose (MC) have been defined [166]. Other formulations described the use of gellan gum [172], and its further combinations with polyethylene glycol (PEG)/sodium lauryl sulfate (SLS) [173] or pullulan/mannitol [171]. In addition, a formulation comprising xanthan gum with sodium alginate/citric acid was also reported [186].

#### 5.1.4. Gelatin-Based Coatings

Gelatin has been applied as individual coating material in order to achieve reduced stickiness and glutinous behavior [168]. Other combinations of gelatin with lubricants [169], sodium alginate/vegetable oil [188], carrageenan/HPMC/starch/polymethacrylate [182], and glycerin/glucose/gum arabic have also been published [189].

#### 5.1.5. Sodium Alginate-Based Coatings

Sodium alginate has been applied as a thickening agent in order to manufacture a coating material that swells and forms a gel upon the uptake of water [162].

#### 5.1.6. Wax-Based Coatings

An anti-adhesive coating of beeswax and talc to obtain good slip properties has been disclosed in a patent [181]. 

#### 5.1.7. Shellac-Based Coatings

A material that is composed of water-soluble shellac has been proposed to contribute for pharmaceutically elegant tablets that enhance swallowability [164]. Another patent described a solution comprising a mixture of shellac/PVP/hydroxypropyl cellulose (HPC)/PEG/sucralose [187]. 

#### 5.1.8. Polyacrylate-Based Coatings

A two-layered polyacrylic acid coating material in combination with sodium carboxymethylcellulose (CMC)/PVP, which forms a viscous surface after absorbing saliva, was suggested [60]. Furthermore, an acrylic acid copolymer formulation has also been described [170].

#### 5.1.9. Polyethylene Oxide-Based Coatings

A polyethylene oxide (PEO) coating has been proposed as lubricious material for pharmaceutical applications. The coating can be applied by dipping the SODF in the coating solution, followed by curing process with ultraviolet light [177].

#### 5.1.10. Carrageenan-Based Coatings

A film composed of carrageenan and trehalose that converted to an easy-to-swallow smooth surface was disclosed in a patent [174]. A complex mixture comprising carrageenan/sodium alginate/xanthan gum/HPMC/crospovidone has been proposed as coating material in order to enhance tablet swallowability [180]. Other combinations, including carrageenan/agar/gelatin, were also reported [183].

#### 5.1.11. Polysaccharide-Based Coatings

A flavored coating solution containing viscous and lubricant materials (e.g., polysaccharides, polyols) that can be applied to SODF by spraying or dipping was previously detailed in a patent record [175]. In addition, a coating gel that was obtained by polymerization and crosslinking of different polysaccharides that contributes to reduced esophageal friction was also suggested [185].

### 5.2. Clinical Evidence of Proposed Coating Compositions for Enhanced Swallowability

Clinical studies involving healthy volunteers have been performed for some of the described coatings compositions. Fluoroscopic measurements with 10 healthy volunteers while using the PVA/carboxyvinyl coating combination provided evidence for the accelerated transit time of the coated SODF as compared to gelatin capsules [161]. The mixture of PVA/polyacrylic acid/glycerin was assessed in a study with five volunteers, which confirmed a good swallowing experience that was provided by the coating [178]. Five healthy volunteers were also enrolled in an in vivo trial that assessed gellan gum/pullulan/mannitol coatings [171]. 

Another clinical study has shown that shellac/PVP/HPC/sucralose-based coatings can reduce the tendency of SODF to adhere into the oral cavity of patients [187]. Improved taste and optimal swallowing experience upon SODF administration was identified through sensory assessments for both the polyacrylic acid/CMC/PVP and acrylic acid copolymer coatings [170,179]. Lastly, a clinical trial with 30 subjects reported an improvement in SODF swallowability though a significant reduction of involuntary gag reflexes for lubricant coatings that are composed of polysaccharides/polyols [175].

## 6. Reflections on Available Administration Aids and Devices to Enhance SODF Swallowability in Older Patient Populations

Two of the identified administration aids/devices are currently marketed as swallowing-enhancing technologies for SODF. These can be sub-grouped into distinct co-administration mechanisms involving SODF suction with jelly vehicles [83], spraying of the SODF and patient’s mouth and/or tongue with lubricants [87], and the manual application of a gelatinous coating onto the SODF before administration [96]. Semi solid vehicles are typically recommended for patients with swallowing issues, as their rheological properties allow for the formation of a bolus that is smooth to swallow and prevents cases of aspiration [82,191]. When embedded into the semi-solid vehicles, the SODF are not recognized as a bulk solid by the patients and they do not directly interfere with their oro-esophageal system, therefore preventing cases of mucosal sticking and gag reflex. Nevertheless, the co-administration with such type of swallowing aids requires the proper handling and it might be limited by the patient’s sip volume, as well as the number of daily doses to be administered, which may limit their use by older patients. 

The swallowing-enhancing properties of the spraying solution (*Pill Glide^®^*) are supported by specific formulation ingredients, namely xanthan gum and glycerin, as their film-forming and plasticizing effects are expected to coat the oral mucosa and the SODF, reducing the friction and improving the swallowing experience for the patient [192,193]. 

The flexible integrity of *MedCoat^®^* conferred by gelatin allows for the manual application of the coating onto the SODF, while the swallowing-enhancing properties of the material are exerted by a combination of the slippery attribute of vegetable oils, the surfactant effect of fatty acid sugar esters, and the saliva stimulation provided by citric acid [194,195]. Moreover, the maltitol and lemon flavor ingredients are expected to increase the palatability and improve the acceptability of the SODF by older patients [196].

The clinical evaluations for the GT packaging and MedCoat^®^ administration aids were directed to older patient populations. The endpoints and assessments instruments varied, according to the type of administration aid tested, with a general use of qualitative scales for swallowing experience being adopted in all studies. A three-step sensory test was used for the GT packaging, which included opening (breaking the film cover), pushing the gel with the fingers, and preference of co-administration with the packaging. On the other hand, the easiness of swallowing and SODF palatability were the endpoints that were reported by the patients during the trials with *MedCoat^®^* [83,88].

## 7. Reflections on Identified Film Coating Materials to Enhance SODF Swallowability in Older Patient Populations

The modification of the surface properties of SODF to improve the swallowing experience for older patients can be achieved with pharmaceutical coatings. The identified coating technologies were mainly focused on water-soluble polymers, in combination with excipients providing additional functions. The swallow-enhancing mechanism for PVA-based coatings is related quick hydration, due to the formation of hydrogen bonds between the saliva water molecules and OH groups in the polymer monomer units [197]. Further combinations of PVA with carboxyvinyl polymers and polyacrylic acid/glycerin will increase the water absorbing and swelling properties of the coating, promoting a gel-forming surface and increasing the slip effect of the SODF in the esophagus [161,198].

The cellulose-based coatings were mainly HPMC-derived, as modified celluloses are predicted to hydrate and uptake water more efficiently, due to the increased hydrophilicity granted by hydroxypropyl groups, contributing to the formation of a gel-like surface in the SODF [199]. Additional combinations of HPMC with ethyl cellulose/PVP are also expected to increase the slip properties of the coating surface, due to a combination of hydrophobic and binder properties, respectively [200]. 

The gelling and emulsifying capabilities of gelatin alone are expected to contribute for a better swallowing experience when administering SODF. However, the gliding properties of gelatin coatings can be further optimized with additives, such as HPMC and polysaccharides, in which their hydroxyl and carboxyl groups will increase the water binding and optimize wettability [201,202].

The swallowing enhancement mechanism for gum-based coating are mainly related to their swelling properties, which are conferred by water binding capacity and quick hydration. The level of water binding can be increased depending on the gum applied in the coating formulation, and higher binding capacity can be achieved with xanthan gum. Guar gum and sodium alginate, as stand-alone coating materials, will present lower binding capabilities and, as such, associations with water-soluble additives, surfactants (e.g., SLS) and saliva promoting agents (e.g., citric acid) will contribute to an improvement in their gliding performances [203]. 

The gelling properties of carrageenan are associated with the presence of anhydro galactose units, with a higher softness and gelling elasticity being achieved for ι-carrageenan, due to its lower content in units when compared to with κ-carrageenan. In addition, further combinations with water-soluble additives are expected to promote the gelling effect of carrageenan [204]. 

The enhancing SODF swallowing experience with wax and shellac-based coatings is associated with the hydrophobic nature of these molecules, as their expected smooth surface will reduce the coefficient of friction and increase slip properties [205]. Last but not least, the fast emulsifying properties of polyacrylates will generate a swellable SODF coating surface when in contact with saliva, and they are expected to entail suitable viscosity for a better swallowing experience. Further combinations with water-soluble additives (e.g., CMC and PVP) will increase the coating water uptake and promote a better SODF gliding surface [206].

Although the majority of the identified coating materials allege to enhance SODF swallowability, their clinical evidence to support such claims is still very limited [207]. Furthermore, the available literature published in recent years has tended to focus more on observational studies to measure overall patient acceptability in older patient populations, rather than investigating SODF characteristics and their critical endpoints for swallowability enhancement. Therefore, the current lack of research on developing relevant evidence on the relationship between the physical characteristics of SODF and their direct correlation to swallowability appears to be the main reason for the limited number of scientific articles that were identified within this literature review [26,147,208,209,210].

Along with the development of technical approaches and solutions, the collection of clinical data for the concerned patient populations will be required in order to confirm the theoretical models underlying the scientific and technical rationale for drug products that make claims of enhanced swallowability or appropriateness for special patient populations. As such, further clinical assessments are required for validating their potential to overcome swallowing issues [126,128,143,158,159].

## 8. Concluding Remarks

Swallowing issues with SODF are being increasingly recognized as a growing health condition throughout healthcare professionals. There is a consensus that the size, shape, color, taste, and mouthfeel have a significant impact on drug product swallowability and acceptance. In order to achieve good compliance, as well as effective, safe, and independent pharmacotherapy, it is important for physicians and pharmaceutical professionals to be informed regarding potential problems that are related to a patient’s inability to swallow SODF, in order to prescribe/dispense suitable drug formulations and/or designs that can better meet the specific needs of each patient [26,32,121,207].

Technologies for improving the swallowability of SODF have been developed and tested throughout the years; nevertheless, these often require preparative steps by the patient and, as such, remain very dependent on user’s handling capabilities. When considering the older, multimorbid, frail, and polymedicated patients; this might further increase the therapeutic complexity and lead to non-compliance or medication errors [211,212,213,214]. It was noticed that all of the clinical assessments were sponsored, or at least supported, by companies owning the swallowing enhancing technology under investigation. Other studies that were financed by public funds or independent research groups comparing different swallowing enhancing technologies with scientific or clinical endpoints were not identified.

More attention has been given to the development of new coating technologies for SODF. A large number of patents claiming new intellectual property were published, disclosing new coating formulations and its relative-preparation methods. The coatings can be applied to oral solid forms and they have been suggested to provide enhanced swallowing experience to both healthy and dysphagic patients. However, clinical evidence confirming the swallowing benefits of the coating formulations in the concerned patient populations are still very limited. In addition, very few of the suggested technologies have been introduced in the market, with evidence of their potential to overcome swallowing issues in the most vulnerable, older patient population being very limited. In this respect, the “gold standard” HPMC coating must still be considered to be state of the art in tablet coating, even though it does not specifically enhance swallowability when compared to other SODF [100,106].

When it comes to older patients with dysphagia, nowadays SODF administration still remains an unresolved challenge within the subject of pharmaceutical technology. Besides the development of technical approaches and solutions, clinical data in the concerned patient populations will be required to confirm the theoretical models underlying the scientific and technical rationales for drug products claiming enhanced swallowability or appropriateness for older patient populations [215,216,217,218].

## Figures and Tables

**Table 1:** Disease-related conditions as predisposition for developing dysphagia [1].

Predisposition	Condition
*Neurologic disorders and stroke*	Cerebral infarction
	Brain-stem infarction
	Intracranial hemorrhage
	Parkinson’s disease
	Multiple sclerosis
	Amyotrophic lateral sclerosis
	Poliomyelitis
	Myasthenia gravis
	Dementia
*Structural lesions*	Thyromegaly
	Cervical hyperostosis
	Congenital web
	Zenker’s diverticulum
	Ingestion of caustic material
	Neoplasm
*Psychiatric disorder*	Psychogenic dysphagia
*Connective tissue diseases*	Polymyositis
	Muscular dystrophy
*Iatrogenic causes*	Surgical resection
	Radiation fibrosis
	Medications

**Table 2:** Medication that may affect swallowing function [1].

Physiological Condition	Class of Drugs
*Sedation, pharyngeal weakness, dystonia*	Benzodiazepines
	Neuroleptics
	Anticonvulsants
*Myopathy*	Corticosteroids
	Lipid-lowering drugs
*Xerostomia*	Anticholinergics
	Antihypertensives
	Antihistamines
	Antipsychotics
	Narcotics
	Anticonvulsants
	Antiparkinsonian agents
	Antineoplastics
	Antidepressants
	Anxiolytics
	Muscle relaxants
	Diuretics
*Inflammation (from tablet irritation)*	Tetracycline
	Doxycycline
	Iron preparations
	Quinidine
	Nonsteroidal anti-inflammatory drugs
	Potassium
*Impaired motility or gastroesophageal reflux*	Anticholinergics
	Calcium channel blockers
	Theophylline
*Esophagitis (related to immunosuppression)*	Corticosteroids

**Table 3:** Scientific articles addressing administration aids to assist swallowability of SODF.

Authors	Title	Year	Reference
Diamond et al.	Experience with a pill-swallowing enhancement aid	2010	[87]
Uloza et al.	A randomized cross-over study to evaluate the swallow-enhancing and taste-masking properties of a novel coating for oral tablets	2010	[88]
Sadamoto et al.	Innovative Tool for Taking Large Pills for the Elderly and Patients with Swallowing Difficulties	2012	[83]

**Table 4:** Patents addressing administration aids to assist swallowability of SODF.

Author(s)	Patent Number	Related Invention	Year	Reference
L.A. Lenk	US2007275053A1	Anti-stick formula delivered by spray process to facilitate swallowing of solid object, such as pill, tablet, capsule or caplet	2007	[89]
Craig et al.	WO2009098520A2	Composition and method for assisting swallowing	2009	[86]
Axelsson et al.	WOUS2018311108	A new coating composition and use thereof	2010	[90]
Guomin et al.	CN103721264A	Gel for assisting swallow of oral solid medicinal preparation	2014	[84]
Morimoto et al.	WO2014064840A1	Device for oral drug administration	2014	[85]
Nappi Bryan	US2018311108A1	Pill coating apparatus and method	2018	[91]

**Table 5:** Inclusion and exclusion criteria used in the review.

Priority Order	Inclusion	Exclusion
1	Oral drug delivery	Other routes for drug delivery
2	Capsules and tablets	Powders, granules, sachets, multiparticulates, effervescent tablets
3	Tablets swallowed intact (e.g., non-dispersible, bulk tablets)	Dispersible tablets (e.g., dispersible, effervescent, orodispersible)
4	Interventions to facilitate swallowing of tablets and capsules	Dosage form manipulations
5	Coatings to enhance swallowing of tablets and capsules	Other functional coatings

**Table 6:** Scientific articles addressing coating materials to enhance the swallowability of SODF.

Authors	Title	Year	Reference
Okabe et al.	Development of an easily swallowed film formulation	2008	[161]
Ito et al.	Investigation of Oral Preparation That Is Expected to Improve Medication Administration: Preparation and Evaluation of Oral Gelling Tablet Using Sodium Alginate	2017	[162]

**Table 7:** Patents addressing new coating materials to enhance swallowability of SODF.

Author(s)	Patent Number	Year	Related Invention	Reference
William N. Clark	US209654A	1878	Improvement in soluble coatings for pills	[163]
Secora et al.	US3390049A	1968	Pharmaceutical tablets coated with wax-free ammonia solubilized water soluble shellac	[164]
John et al.	US4302440A	1981	Easily-swallowed aspirin tablet thinly-coated with HPMC and aqueous spray-coating preparation	[165]
Motoaki Sato	JPS61161215A	1986	Method of making solid material easily swallowable	[166]
Tencza et al.	CA1217140A	1987	Thin film coated tablets	[167]
Becker et al.	US5114720A	1992	Gelatin coated tablets and method for producing same	[168]
S. Imanishi	JPH09104621A	1997	Medicine coated with gelatinizing agent, lubricating agent and lubricant	[169]
Peter Gruber	WO9806385A1	1998	Easy to swallow oral medicament composition	[170]
Nitsuto et al.	JP2002275054A	2002	Easily administrable solid preparation	[171]
Flanagan et al.	US6395298B1	2002	Gellan gum tablet coating	[172]
Flanagan et al.	US6635282B1	2003	Gellan gum tablet film coating	[173]
Tsukioka et al.	JP2007070344A	2007	Internal medicine	[174]
Jerry Robertson	US20070259038A1	2007	Solid medicament dosage form consumption aid	[175]
Kawasumi et al.	JP2007015950A	2007	Easily-swallowable film-coated preparations containing antacids	[176]
Eramo Lincoln	US2007243246A1	2007	Lubricious coatings for pharmaceutical applications	[177]
Kata et al.	JP2009120497A	2009	Film for assisting deglutition and method for producing the same	[178]
Kata et al.	JP2010120877A	2010	Oral administration preparation	[179]
Fujioka et al.	JP2011195569A	2011	Easily swallowable tablet	[180]
Chen et al.	TW 201121586A	2011	Oral tablet	[181]
Joel Waldman	WO2012024360A2	2012	Tablet sleeve for improved performance	[182]
Yang et al.	CN102652738A	2012	Novel medicinal outer wrapper facilitating swallowing	[183]
Sugiura et al.	CN102361652A	2012	Adhesion preventing composition, solid preparation and method for producing the same	[184]
Li et al.	CN102430124A	2012	Pill coating with ultralow friction coefficient and preparation method	[185]
Mizuhara et al.	JP2014227391A	2014	Water-swellable laminated film and swallowable substance-coated body	[186]
Takano et al.	JP2014189547A	2014	Swallowable film-coated cover for oral drug delivery	[187]
Bao Yinjian	CN108543072A	2018	Coating composition and related used thereof	[188]
Bao Yinjian	CN108578704A	2018	Composition used for swallowing and relevant applications of composition	[189]
Jeffrey et al.	US2018036413A1	2018	Easy to swallow coatings and substrates coated therewith	[190]

## Data Availability

Not applicable.

## Supplementary Materials

Supplementary materials are available online at https://www.mdpi.com/1999-4923/13/1/32/s1.
