# Supplementary material for: Better Medicines for Older Patients: Considerations between Patient Characteristics and Solid Oral Dosage Form Designs to Improve Swallowing Experience
Source: Pharmaceutics. 2020 Dec 28;13(1):32. doi: 10.3390/pharmaceutics13010032 (PMC7824227; doi:10.3390/pharmaceutics13010032)
Supplement: Supplementary file 1 [file pharmaceutics-13-00032-s001.pdf]

pharmaceutics

Supplementary Materials: Better Medicines for Older Patients: Considerations between Patient Characteristics and Solid Oral Dosage form Designs to Improve Swallowing Experience

Nélio Drumond and Sven Stegemann

A: List of keywords applied during the search

DELIVERY
- oral medication
- drug delivery
- oral delivery
- oral administration
- oral route
- oral drug delivery
- drug delivery techniques
- drug delivery technology
- drug delivery systems
- drug modifications
- patient-centered
- patient-centered drug delivery
- drug intake
- drug design

PHATOLOGIES/SWALLOWING PRBLEMS
- swallowing disorders
- swallowing issues
- impaired swallowing
- impaired swallowability
- difficult to swallow
- swallowing difficulties
- swallow
- swallowing problems
- swallowing
- swallowability
- dysphagia
- dysphasic
- dysphasic patients
- dysphasic people
- patient experiences
- patient experience
- aspiration
- aspiration pneumonia
- silent aspiration
- pneumonia
- esophagitis
- oropharyngeal dysphagia
- malnutrition
- food bolus
- bolus viscosity
- oral residence time
- residence time

- residence in esophagus
- sticking in esophagus
- esophagus sticking
- deglution problems
- deglutition
- deglutition disorders
- efficacy of swallowing
- safety of swallowing

POPULATION
- human
- elderly
- patient
- old population
- old people
- old
- ageing
- geriatric
- geriatric population
- special populations
- special patients
-special patient populations
- older patients
- diseases
- morbidities
- dementia
- Parkinson
- stroke

CLINICAL EXAMS FOR ASSESSING SWALLOWING ISSUES
- electromyogram
- nasopharyngeal endoscopic evaluation
- nasopharyngeal endoscopy
- surface electromyogram
- surface electromyogram measurement
- respiratory monitoring
- scintigraphy
- gamma scintigraphy
- video fluoroscopy
- fluoroscopy
- fluoroscopic study

ADHESION/ADHESION STUDIES
- adhesion studies
- mucosal surface
- oesophageal
- oesophagus
- esophagus
- esophageal
- oesophageal transit
- esophageal transit
- human oesophagus
- porcine oesophagus
- porcine esophagus
- mucosa

- esophageal mucosa
- oesophageal mucosa
- adherence
- adhesion
- oesophageal adhesion
- esophageal adhesion
- film adhesion
- force of adhesion
- bioadhesion
- mucoadhesion
- adhesion studies
- mucoadhesive
- mucoadhesive drug delivery systems
- mucoadhesion assessment
- mucoadhesion assessment techniques
- mucoadhesion techniques
- mucoadhesive film
- mucoadhesive polymers
- adhesive coating
- non-adhesive coating
- intestinal mucosa
- tensile strength tester

TESTING
- viscosity
- rheology
- solubility
- permeability
- film brittleness
- stretch-ability
- surface tension
- rheological measurements
- tensile strength
- ellipsometry
- non-destructive testing
- optical coherence tomography
- process analytical technology
- surface free energy
- melt viscosity
- contact angle
- surface energy
- coating efficiency
- atomic force microscopy
- AFM
- interparticle interaction
- detachment force
- elongation
- adhesive toughness
- surface energy
- surface energetics

DOSAGE FORMS
- liquid
- suspension
- semi-solid

- pellets
- tablet
- pill
- capsule
- color
- shape
- dimension
- flat
- convex
- round
- oblong
- sharp
- gloss
- gloss finish
- large tablets
- oral dosage forms
- oral dosage form
- solid oral dosage
-solid oral dosage form
- coating
- size
- generic
- therapeutic equivalence
- tablet surface
- dosage forms
- pharmaceutical dosage forms

<u>FORMULATION/EXCIPIENTS</u>

- film-coat
- film-coating
- film coating
- aqueous film coating
- film formulation
- gelation
- swelling
- coating
- coat
- polymer
- tablet coating
- pharmaceutical engineering
- modeling
- coating process parameters
- coating technologies
- coating material
- coating composition
- coating formulation
- polymeric film coating
- continuous processing
- continuous coating
- process analytical technologies
- wax
- waxes
- main polymer
- solubilizer

- binder
- disintegrants
- antiadherents
- lubricants
- glidant
- glidants
- glidant properties
- surfactant
- emulsifier
- humectant
- plasticizer
- additive
- additives
- colorants
- sweeteners
- taste suppressants
- taste potentiators
- solvent
- polymer-plasticizer interaction
- glass transition

COATING TECHNOLOGY
- pan coating
- pan coater
- perforated pan coater
- accela-cota coating
- accela-cota
- fluidized bed coating
- fluid bed coater
- hot-melt coating
- fluidized bed
- scale-up
- controlled release
- dry powder coating
- dry coating
- rotor granulation
- melt extrusion
- film formation
- rotary fluid bed
- material application
- moisture protection
- coating process

PROCESS VARIABLES IN TABLET COATING
- processing
- inlet bed temperature
- outlet bed temperature
- humidity
- relative humidity
- atomization
- air pressure
- atomization air pressure
- pan speed
- spray rate
- liquid spray rate

- gun nozzle
- spray gun
- solid content of coating solution
- nozzle-to-bed distance
- droplet size
- drying time
- tablet bed

<u>COATING</u>
- sugar coating
- film coating
- press coating
- enteric coating
- pH dependent coating
- moisture protection
- gastric protection
- subcoating
- taste masking
- lipid coating
- delayed dissolution

<u>COATING APPEARENCE</u>
- color
- color variation
- sticking
- twinning
- cracking
- capping
- lamination
- broken tablet
- orange peel
- fibers
- black spots
- oil spots
- picking
- roughness
- blistering
- catering

<u>RELEASE</u>
- controlled release
- sustained release
- immediate release

<u>GENERAL</u>
- slippery
- fast diffusion
- diffusion
- glidant
- glidant properties
- slippery surface
- slippery coating
- easy to swallow
- patient-friendly
- swallowing enhancement
- non-adhesive

- poor bioadhesiveness
- stickiness
- sticking coating
- surface treatment
- swallowing enhancing surface
- adherence to medication
- adherence
- fast transport

B: Example of a search strategy script

=> fil ca

=> act durm3/L

L1 (474288)SEA FILE=CA ORAL MEDICATION OR DRUG DELIVERY OR ORAL DELIVERY O

L2 (426928)SEA FILE=CA DRUG DELIVERY SYSTEMS OR DRUG MODIFICATIONS OR PATI

L3 (224)SEA FILE=CA SWALLOWING DISORDERS OR SWALLOWING ISSUES OR IMPAIR

L4 (5011)SEA FILE=CA SWALLOW OR SWALLOWING PROBLEMS OR SWALLOWING OR SWA

L5 (8991)SEA FILE=CA DYSPHASIC PATIENTS OR DYSPHASIC PEOPLE OR PATIENT E

L6 (44742)SEA FILE=CA SILENT ASPIRATION OR PNEUMONIA OR ESOPHAGITIS OR OR

L7 (46573)SEA FILE=CA ORAL RESIDENCE TIME OR RESIDENCE TIME OR RESIDENCE

L8 (109)SEA FILE=CA DEGLUTITION OR DEGLUTITION DISORDERS OR EFFICACY OF

L9 (0)SEA FILE=CA HUMANS/CT AND (ELDERLY/TI OR PATIENT#/TI OR OLD POP

L10 (3776)SEA FILE=CA GERIATRIC POPULATION OR SPECIAL POPULATIONS OR SPEC

L11 (114786)SEA FILE=CA DISEASES/TI OR MORBIDITIES/TI OR DEMENTIA/TI OR PAR

L12 (929)SEA FILE=CA ELECTROMYOGRAM OR NASOPHARYNGEAL ENDOSCOPIC EVALUAT

L13 (11782)SEA FILE=CA SURFACE ELECTROMYOGRAM MEASUREMENT OR RESPIRATORY M

L14 (1158)SEA FILE=CA VIDEO FLUOROSCOPY OR FLUOROSCOPY OR FLUOROSCOPIC ST

L15 (40485)SEA FILE=CA ADHESION STUDIES OR MUCOSAL SURFACE OR OESOPHAGEAL

L16 (57)SEA FILE=CA OESOPHAGEAL TRANSIT OR ESOPHAGEAL TRANSIT OR HUMAN

L17 (110004)SEA FILE=CA MUCOSA/TI OR ESOPHAGEAL MUCOSA/TI OR OESOPHAGEAL MU

L18 (26954)SEA FILE=CA ESOPHAGEAL ADHESION OR FILM ADHESION OR FORCE OF AD

L19 (4278)SEA FILE=CA ADHESION STUDIES OR MUCOADHESIVE OR MUCOADHESIVE DR

L20 (78)SEA FILE=CA MUCOADHESION ASSESSMENT TECHNIQUES OR MUCOADHESION

L21 (21108)SEA FILE=CA MUCOADHESIVE POLYMERS OR ADHESIVE COATING OR NON-AD

L22 (975167)SEA FILE=CA VISCOSITY OR RHEOLOGY OR SOLUBILITY OR PERMEABILITY

L23 (328962)SEA FILE=CA RHEOLOGICAL MEASUREMENTS OR TENSILE STRENGTH OR ELL

L24 (116589)SEA FILE=CA PROCESS ANALYTICAL TECHNOLOGY OR SURFACE FREE ENERG

L25 (111900)SEA FILE=CA COATING EFFICIENCY OR ATOMIC FORCE MICROSCOPY OR AF

L26 (306649)SEA FILE=CA ELONGATION OR ADHESIVE TOUGHNESS OR SURFACE ENERGY

L27 (2510048)SEA FILE=CA LIQUID OR SUSPENSION OR SEMI-SOLID OR PELLETS OR TA

L28 (48700)SEA FILE=CA DIMENSION/TI OR FLAT/TI OR CONVEX/TI OR ROUND/TI OR

L29 (236762)SEA FILE=CA ORAL DOSAGE FORM/TI OR SOLID ORAL DOSAGE/TI OR COAT

L30 (67744)SEA FILE=CA THERAPEUTIC EQUIVALENCE OR TABLET SURFACE OR DOSAGE

L31 (27285)SEA FILE=CA FILM-COAT/TI OR FILM-COATING/TI OR FILM COATING/TI

L32 (50474)SEA FILE=CA COATING TECHNOLOGIES OR COATING MATERIAL OR COATING

L33 (156081)SEA FILE=CA CONTINUOUS PROCESSING OR CONTINUOUS COATING OR PROC

L34 (373161)SEA FILE=CA MAIN POLYMER OR SOLUBILIZER OR BINDER OR DISINTEGR

L35 (55673)SEA FILE=CA GLIDANT/TI OR GLIDANTS/TI OR GLIDANT PROPERTIES/TI

L36 (142782)SEA FILE=CA POLYMER-PLASTICIZER INTERACTION OR GLASS TRANSITION

L37 (1157)SEA FILE=CA PAN COATING OR PAN COATER OR PERFORATED PAN COATER

L38 (164449)SEA FILE=CA FLUID BED COATER OR HOT-MELT COATING OR FLUIDIZED B

L39 (37914)SEA FILE=CA DRY POWDER COATING OR DRY COATING OR ROTOR GRANULAT

L40 (286463)SEA FILE=CA ROTARY FLUID BED OR MATERIAL APPLICATION OR MOISTUR

L41 (196584)SEA FILE=CA PROCESSING/TI OR INLET BED TEMPERATURE/TI OR OUTLET

L42 (40348)SEA FILE=CA ATOMIZATION OR AIR PRESSURE OR ATOMIZATION AIR PRES

L43 (3402)SEA FILE=CA LIQUID SPRAY RATE OR GUN NOZZLE OR SPRAY GUN OR SOL

L44 (16131)SEA FILE=CA DROPLET SIZE OR DRYING TIME OR TABLET BED

L45 (16273)SEA FILE=CA SUGAR COATING OR FILM COATING OR PRESS COATING OR E

L46 (2847)SEA FILE=CA GASTRIC PROTECTION OR SUBCOATING OR TASTE MASKING O

L47 (168108)SEA FILE=CA COLOR/TI OR COLOR VARIATION/TI OR STICKING/TI OR TW

L48 (150883)SEA FILE=CA BROKEN TABLET/TI OR ORANGE PEEL/TI OR FIBERS/TI OR

L49 (1494)SEA FILE=CA SLIPPERY COATING OR EASY (W) SWALLOW OR PATIENT-FRI

L50 (40417)SEA FILE=CA ADHERENCE (W) MEDICATION OR ADHERENCE OR FAST TRANS

L51 (0)SEA FILE=CA (L3 OR L4 OR L5 OR L6 OR L7 OR L8) AND (SWALLOWING/

L52 (17)SEA FILE=CA ((HUMANS/CT AND (ELDERLY/TI OR PATIENT#/TI OR OLD P

L53 (13857)SEA FILE=CA (L12 OR L13 OR L14)

L54 (17)SEA FILE=CA ((HUMANS/CT AND (ELDERLY/TI OR PATIENT#/TI OR OLD P

L55 (821)SEA FILE=CA ((L3 OR L4 OR L5 OR L6 OR L7 OR L8)) AND ((ORAL MED

L56 (4)SEA FILE=CA L55 AND ((L9 OR L10 OR L11))

L57 (5)SEA FILE=CA L55 AND ((L9 OR L10 OR L11) OR AGED/TI)

L58 (20)SEA FILE=CA ((SWALLOWING DISORDERS/TI OR SWALLOWING ISSUES/TI O

L59 (25)SEA FILE=CA L58 OR L57

=> s (l1 or l2) and (l3-l8) and (L37/ti or L38/ti or L39/ti or L40/ti)

```
 344891 ORAL
  36560 MEDICATION
    478 ORAL MEDICATION
          (ORAL(W)MEDICATION)
1362940 DRUG
 544390 DELIVERY
 410257 DRUG DELIVERY
          (DRUG(W)DELIVERY)
 344891 ORAL
 544390 DELIVERY
   4202 ORAL DELIVERY
          (ORAL(W)DELIVERY)
 344891 ORAL
 646996 ADMINISTRATION
  73809 ORAL ADMINISTRATION
          (ORAL(W)ADMINISTRATION)
 344891 ORAL
 169104 ROUTE
   3816 ORAL ROUTE
          (ORAL(W)ROUTE)
 344891 ORAL
1362940 DRUG
 544390 DELIVERY
  58926 ORAL DRUG DELIVERY
          (ORAL(W)DRUG(W)DELIVERY)
1362940 DRUG
 544390 DELIVERY
 733363 TECHNIQUES
     70 DRUG DELIVERY TECHNIQUES
          (DRUG(W)DELIVERY(W)TECHNIQUES)
1362940 DRUG
 544390 DELIVERY
```

261998 TECHNOLOGY
55 DRUG DELIVERY TECHNOLOGY
(DRUG(W)DELIVERY(W)TECHNOLOGY)
1362940 DRUG
544390 DELIVERY
1956057 SYSTEMS
383063 DRUG DELIVERY SYSTEMS
(DRUG(W)DELIVERY(W)SYSTEMS)
1362940 DRUG
175364 MODIFICATIONS
32 DRUG MODIFICATIONS
(DRUG(W)MODIFICATIONS)
385899 PATIENT
74521 CENTERED
150 PATIENT-CENTERED
(PATIENT(W)CENTERED)
385899 PATIENT
74521 CENTERED
1362940 DRUG
544390 DELIVERY
0 PATIENT-CENTERED DRUG DELIVERY
(PATIENT(W)CENTERED(W)DRUG(W)DELIVERY)
1362940 DRUG
183997 INTAKE
1347 DRUG INTAKE
(DRUG(W)INTAKE)
1362940 DRUG
796788 DESIGN
45487 DRUG DESIGN
(DRUG(W)DESIGN)
2488 SWALLOWING
370988 DISORDERS
29 SWALLOWING DISORDERS
(SWALLOWING(W)DISORDERS)
2488 SWALLOWING
111330 ISSUES
2 SWALLOWING ISSUES
(SWALLOWING(W)ISSUES)
150345 IMPAIRED
2488 SWALLOWING
17 IMPAIRED SWALLOWING
(IMPAIRED(W)SWALLOWING)
150345 IMPAIRED
12 SWALLOWABILITY
0 IMPAIRED SWALLOWABILITY
(IMPAIRED(W)SWALLOWABILITY)
205079 DIFFICULT
1383 SWALLOW
39 DIFFICULT TO SWALLOW
(DIFFICULT(1W)SWALLOW)
2488 SWALLOWING
65529 DIFFICULTIES
142 SWALLOWING DIFFICULTIES
(SWALLOWING(W)DIFFICULTIES)

1383 SWALLOW
2488 SWALLOWING
355945 PROBLEMS
37 SWALLOWING PROBLEMS
(SWALLOWING(W)PROBLEMS)
2488 SWALLOWING
12 SWALLOWABILITY
1540 DYSPHAGIA
13 DYSPHASIC
13 DYSPHASIC
1168810 PATIENTS
4 DYSPHASIC PATIENTS
(DYSPHASIC(W)PATIENTS)
13 DYSPHASIC
67653 PEOPLE
0 DYSPHASIC PEOPLE
(DYSPHASIC(W)PEOPLE)
385899 PATIENT
29318 EXPERIENCES
51 PATIENT EXPERIENCES
(PATIENT(W)EXPERIENCES)
385899 PATIENT
97406 EXPERIENCE
58 PATIENT EXPERIENCE
(PATIENT(W)EXPERIENCE)
8878 ASPIRATION
8878 ASPIRATION
28955 PNEUMONIA
281 ASPIRATION PNEUMONIA
(ASPIRATION(W)PNEUMONIA)
18817 SILENT
8878 ASPIRATION
6 SILENT ASPIRATION
(SILENT(W)ASPIRATION)
28955 PNEUMONIA
3415 ESOPHAGITIS
2165 OROPHARYNGEAL
1540 DYSPHAGIA
18 OROPHARYNGEAL DYSPHAGIA
(OROPHARYNGEAL(W)DYSPHAGIA)
12572 MALNUTRITION
693345 FOOD
25629 BOLUS
66 FOOD BOLUS
(FOOD(W)BOLUS)
25629 BOLUS
511545 VISCOSITY
3 BOLUS VISCOSITY
(BOLUS(W)VISCOSITY)
344891 ORAL
59493 RESIDENCE
3152623 TIME
4 ORAL RESIDENCE TIME
(ORAL(W)RESIDENCE(W)TIME)

59493 RESIDENCE
3152623 TIME
46573 RESIDENCE TIME
(RESIDENCE(W)TIME)
59493 RESIDENCE
32168 ESOPHAGUS
0 RESIDENCE IN ESOPHAGUS
(RESIDENCE(1W)ESOPHAGUS)
20829 STICKING
32168 ESOPHAGUS
0 STICKING IN ESOPHAGUS
(STICKING(1W)ESOPHAGUS)
32168 ESOPHAGUS
20829 STICKING
0 ESOPHAGUS STICKING
(ESOPHAGUS(W)STICKING)
1 DEGLUTION
355945 PROBLEMS
0 DEGLUTION PROBLEMS
(DEGLUTION(W)PROBLEMS)
108 DEGLUTITION
108 DEGLUTITION
370988 DISORDERS
3 DEGLUTITION DISORDERS
(DEGLUTITION(W)DISORDERS)
311451 EFFICACY
2488 SWALLOWING
0 EFFICACY OF SWALLOWING
(EFFICACY(1W)SWALLOWING)
314246 SAFETY
2488 SWALLOWING
1 SAFETY OF SWALLOWING
(SAFETY(1W)SWALLOWING)
6274 PAN/TI
232674 COATING/TI
43 PAN COATING/TI
((PAN(W)COATING)/TI)
6274 PAN/TI
893 COATER/TI
13 PAN COATER/TI
((PAN(W)COATER)/TI)
2980 PERFORATED/TI
6274 PAN/TI
893 COATER/TI
1 PERFORATED PAN COATER/TI
((PERFORATED(W)PAN(W)COATER)/TI)
6 ACCELA/TI
45 COTA/TI
232674 COATING/TI
0 ACCELA-COTA COATING/TI
((ACCELA(W)COTA(W)COATING)/TI)
6 ACCELA/TI
45 COTA/TI
4 ACCELA-COTA/TI

((ACCELA(W)COTA)/TI)
41135 FLUIDIZED/TI
68069 BED/TI
232674 COATING/TI
198 FLUIDIZED BED COATING/TI
((FLUIDIZED(W)BED(W)COATING)/TI)
136027 FLUID/TI
68069 BED/TI
893 COATER/TI
11 FLUID BED COATER/TI
((FLUID(W)BED(W)COATER)/TI)
113842 HOT/TI
55602 MELT/TI
232674 COATING/TI
128 HOT-MELT COATING/TI
((HOT(W)MELT(W)COATING)/TI)
41135 FLUIDIZED/TI
68069 BED/TI
32533 FLUIDIZED BED/TI
((FLUIDIZED(W)BED)/TI)
96208 SCALE/TI
70637 UP/TI
3434 SCALE-UP/TI
((SCALE(W)UP)/TI)
116691 CONTROLLED/TI
159623 RELEASE/TI
13709 CONTROLLED RELEASE/TI
((CONTROLLED(W)RELEASE)/TI)
66293 DRY/TI
148173 POWDER/TI
232674 COATING/TI
48 DRY POWDER COATING/TI
((DRY(W)POWDER(W)COATING)/TI)
66293 DRY/TI
232674 COATING/TI
184 DRY COATING/TI
((DRY(W)COATING)/TI)
5425 ROTOR/TI
7715 GRANULATION/TI
1 ROTOR GRANULATION/TI
((ROTOR(W)GRANULATION)/TI)
55602 MELT/TI
25688 EXTRUSION/TI
664 MELT EXTRUSION/TI
((MELT(W)EXTRUSION)/TI)
321218 FILM/TI
485917 FORMATION/TI
6566 FILM FORMATION/TI
((FILM(W)FORMATION)/TI)
16979 ROTARY/TI
136027 FLUID/TI
68069 BED/TI
6 ROTARY FLUID BED/TI
((ROTARY(W)FLUID(W)BED)/TI)

372313 MATERIAL/TI
496228 APPLICATION/TI
141 MATERIAL APPLICATION/TI
((MATERIAL(W)APPLICATION)/TI)
41838 MOISTURE/TI
85520 PROTECTION/TI
69 MOISTURE PROTECTION/TI
((MOISTURE(W)PROTECTION)/TI)
232674 COATING/TI
602104 PROCESS/TI
4890 COATING PROCESS/TI
((COATING(W)PROCESS)/TI)
L60 96 (L1 OR L2) AND ((L3 OR L4 OR L5 OR L6 OR L7 OR L8)) AND ((PAN COATING/TI OR PAN COATER/TI OR PERFORATED PAN COATER/TI OR ACCEL
A-COTA COATING/TI OR ACCELA-COTA/TI OR FLUIDIZED BED COATING/TI)
OR (FLUID BED COATER/TI OR HOT-MELT COATING/TI OR FLUIDIZED
BED/TI OR SCALE-UP/TI OR CONTROLLED RELEASE/TI) OR (DRY POWDER
COATING/TI OR DRY COATING/TI OR ROTOR GRANULATION/TI OR MELT
EXTRUSION/TI OR FILM FORMATION/TI) OR (ROTARY FLUID BED/TI OR
MATERIAL APPLICATION/TI OR MOISTURE PROTECTION/TI OR COATING
PROCESS/TI))

=> s l60 and swall?/Ti
884 SWALL?/TI
L61 0 L60 AND SWALL?/TI

=> s l60 and (l31 or l32 or l33 or l34 or l35 or l36)
321218 FILM/TI
10016 COAT/TI
20 FILM-COAT/TI
((FILM(W)COAT)/TI)
321218 FILM/TI
232674 COATING/TI
1905 FILM-COATING/TI
((FILM(W)COATING)/TI)
321218 FILM/TI
232674 COATING/TI
1905 FILM COATING/TI
((FILM(W)COATING)/TI)
230090 AQUEOUS/TI
321218 FILM/TI
232674 COATING/TI
55 AQUEOUS FILM COATING/TI
((AQUEOUS(W)FILM(W)COATING)/TI)

```
 321218 FILM/TI
  47635 FORMULATION/TI
     51 FILM FORMULATION/TI
          ((FILM(W)FORMULATION)/TI)
   7882 GELATION/TI
  17463 SWELLING/TI
1248683 COATING
 134198 TECHNOLOGIES
    691 COATING TECHNOLOGIES
          (COATING(W)TECHNOLOGIES)
1248683 COATING
2405662 MATERIAL
  38962 COATING MATERIAL
          (COATING(W)MATERIAL)
1248683 COATING
 899138 COMPOSITION
   9097 COATING COMPOSITION
          (COATING(W)COMPOSITION)
1248683 COATING
 262843 FORMULATION
   2126 COATING FORMULATION
          (COATING(W)FORMULATION)
 352272 POLYMERIC
1529251 FILM
1248683 COATING
     27 POLYMERIC FILM COATING
          (POLYMERIC(W)FILM(W)COATING)
 594589 CONTINUOUS
 880973 PROCESSING
   1593 CONTINUOUS PROCESSING
          (CONTINUOUS(W)PROCESSING)
 594589 CONTINUOUS
1248683 COATING
   1529 CONTINUOUS COATING
          (CONTINUOUS(W)COATING)
3835271 PROCESS
 144428 ANALYTICAL
 134198 TECHNOLOGIES
     17 PROCESS ANALYTICAL TECHNOLOGIES
          (PROCESS(W)ANALYTICAL(W)TECHNOLOGIES)
 114486 WAX
  92587 WAXES
 872117 MAIN
1675559 POLYMER
    667 MAIN POLYMER
          (MAIN(W)POLYMER)
   5419 SOLUBILIZER
 266445 BINDER
   6246 DISINTEGRANTS
     41 ANTIADHERENTS
 100837 LUBRICANTS
     29 GLIDANT/TI
     20 GLIDANTS/TI
     29 GLIDANT/TI
```

```
      1107362 PROPERTIES/TI
            2 GLIDANT PROPERTIES/TI
                ((GLIDANT(W)PROPERTIES)/TI)
        46095 SURFACTANT/TI
         4131 EMULSIFIER/TI
          307 HUMECTANT/TI
         5178 PLASTICIZER/TI
      1675559 POLYMER
        73259 PLASTICIZER
      1370032 INTERACTION
           29 POLYMER-PLASTICIZER INTERACTION
                (POLYMER(W)PLASTICIZER(W)INTERACTION)
      1074984 GLASS
      1410258 TRANSITION
       142760 GLASS TRANSITION
                (GLASS(W)TRANSITION)
L62            15 L60 AND (L31 OR L32 OR L33 OR L34 OR L35 OR L36)


=> d l62 ti 1-15
```
